# Establishing the baseline level of repetitive element expression in the human cortex

**DOI:** 10.1186/1471-2164-12-495

**Published:** 2011-10-10

**Authors:** Svitlana Tyekucheva, Robert H Yolken, W Richard McCombie, Jennifer Parla, Melissa Kramer, Sarah J Wheelan, Sarven Sabunciyan

**Affiliations:** 1 Department of Biostatistics and Computational Biology, Dana-Farber Cancer Institute, 450 Brookline Ave, Boston, 02115, USA; 2 Department of Biostatistics, Harvard School of Public Health, 677 Huntington Ave, Boston, 02115, USA; 3 Department of Pediatrics, Johns Hopkins University School of Medicine, 600 N. Wolfe Street, Baltimore, 21287, USA; 4 Stanley Institute for Cognitive Genomics, Cold Spring Harbor Laboratory, 1 Bungtown Road, Cold Spring Harbor, 11724, USA; 5 Department of Oncology, Sidney Kimmel Comprehensive Cancer Center, Johns Hopkins University School of Medicine, 550 N. Broadway, Baltimore, 21205, USA; 6 Department of Biostatistics, Johns Hopkins Bloomberg School of Public Health, 615 N. Wolfe Street, Baltimore, 21205, USA; 7 Department of Molecular Biology and Genetics, Johns Hopkins University School of Medicine, 725 N. Wolfe Street, Baltimore, 21205, USA

## Abstract

**Background:**

Although nearly half of the human genome is comprised of repetitive sequences, the expression profile of these elements remains largely uncharacterized. Recently developed high throughput sequencing technologies provide us with a powerful new set of tools to study repeat elements. Hence, we performed whole transcriptome sequencing to investigate the expression of repetitive elements in human frontal cortex using postmortem tissue obtained from the Stanley Medical Research Institute.

**Results:**

We found a significant amount of reads from the human frontal cortex originate from repeat elements. We also noticed that Alu elements were expressed at levels higher than expected by random or background transcription. In contrast, L1 elements were expressed at lower than expected amounts.

**Conclusions:**

Repetitive elements are expressed abundantly in the human brain. This expression pattern appears to be element specific and can not be explained by random or background transcription. These results demonstrate that our knowledge about repetitive elements is far from complete. Further characterization is required to determine the mechanism, the control, and the effects of repeat element expression.

## Background

Roughly half of the human genome is comprised of repetitive elements; these elements range from the 6Kb LINE1 to micro and minisatellites [1-4]. The biological role of repetitive elements is not known and in general they are believed to be nonfunctional sequences. Some are remnants of ancient germline infections and transposition events [3,5]. These include the human endogenous retroviruses which at one time presumably existed as infectious exogenous agents [6]. Long and Short Interspersed Nuclear Elements (LINE/SINE) are active retrotransposons which continually insert themselves into the human genome, though the vast majority of the elements have mutated beyond function [3,7,8].

Endogenous retroviruses and transposons are largely inactive either through DNA methylation or histone modifications [9,10], or because they have accumulated mutations over time. However, active transposition by LINEs does occur in humans and occasionally causes disease when the insertion occurs in an exon [8], splice site, or regulatory region [11]. Insertion of L1 elements in the antisense orientation can also truncate transcripts by creating new polyadenylation sites (a phenomenon known as gene breaking)[12]. Repetitive elements alter gene expression by introducing promoter regions near genes, or through many other documented mechanisms [13-15]. It has been suggested that repetitive elements contribute to disease pathology by acting as superantigens or by causing an auto-immune response through molecular mimicry, but these hypotheses are largely speculative [16-18]. Surprisingly, certain endogenous retrovirus proteins have acquired beneficial functions in their hosts. For example, the HERV-W env gene, also known as human syncytin-1, contributes to trophoblast cell fusion during placental development [19]. Additionally, the expression of endogenous retroviral and retrotranposon sequences is hypothesized to play a role in preventing infections by exogenous retroviral agents [6]. However, evidence for this theory is tenuous and the current dogma maintains that repeat elements need to be silenced and selected against because they have adverse effects.

Even if the expression of repeat elements is not harmful, repetitive elements have no known function. Because of this, microarray designs generally exclude repeats, both to conserve probes for known functional elements and to avoid having probes whose genomic position is necessarily unknown. Hence, the extent of repeat element expression remains largely unknown. However, there is evidence that certain repetitive elements are expressed at the RNA level in adult somatic cells and some are even translated into proteins [20,21].

High throughput sequencing (HTS) allows the entire transcriptome of a sample to be sequenced in an unbiased manner and is a good method for analyzing genome wide repetitive element expression. In a HTS experiment, gene expression is determined by counting the number of reads which are present from a particular transcript and normalizing over the total number of reads observed. Two recent papers used this approach to assess the level of repeat elements expression and found repetitive elements to be more widely expressed than previously thought [22,23]. One of the papers examined repeat expression in the cerebellum, an area mainly involved in motor movements, and reported higher than expected levels of Alu element expression [22]. However, the authors concluded that expression of other repeat elements was due to "transcriptional background." Indeed, earlier studies suggest that a huge proportion of the human genome is transcribed [24] and transcripts of repetitive elements could be derived from such "promiscuous transcription." We performed deep transcriptome sequencing on the frontal cortex of ten different human postmortem brains and aligned the reads from these samples to all repetitive regions in the human genome. The frontal cortex is involved in complex cognitive functions and abnormalities in this cortical area are associated with various psychiatric illnesses. Consistent with earlier studies, we found that a large proportion of all aligned reads mapped to repeat elements. Unlike previous studies which sequenced pooled samples, we sequenced our samples independently and found that repeat expression patterns are stable between individuals. We also found that Alu element transcripts are expressed at disproportionately high levels whereas truncated LINE elements are expressed at levels much lower than expected. Hence we conclude that the expression of repeat elements is not a side effect of promiscuous, random transcription.

## Results

### Repetitive Elements are expressed in the frontal cortex

In order to determine the expression level of repetitive elements in cortical tissue, mRNA was extracted from 10 post mortem frontal cortex samples, converted to cDNA and paired end HTS libraries were constructed. Initially, four lanes of paired end sequencing was performed on three samples (RUN1 and RUN2 Additional File 1: Table S1) on an Illumina Genome Analyzer. In order to increase our sample size, we sequenced an additional seven samples (RUN3 Additional File 1: Table S1) on an Illumina HiSeq 2000. We aligned the sequencing data from these runs to a human repeats database that we constructed. The database contains the reference genome (NCBI36/hg18 build) sequence of every annotated repeat region in repBase with a 50 bp flank on each side of the repeat. In this way, we could be reasonably certain that a short read that did not align to our database was not derived from a repetitive sequence. The flanking sequences were included so that a short read that spanned the junction of a repeat element with unique genomic DNA would not be missed. We avoided simply aligning the sequencing reads to the consensus sequences for the repeat classes since transcripts originating from older or slightly divergent members of a class of repetitive elements may be missed using this approach, and many of the consensus sequences contain ambiguity characters. Using this custom database has several advantages over simply aligning the reads to the genome assembly, and then selecting repeats that intersect with the repeats annotations. First, our method allows us to include repeats annotated on the "random" chromosomes. Second, we are able to capture reads that otherwise might align to the part of the assembly that is repeat-like, but was not annotated as a repeat in RepBase. When we map directly onto our repeats database, such reads will align to the closest member of the appropriate repeat family and won't be excluded from the analysis.

**Figure 1:**
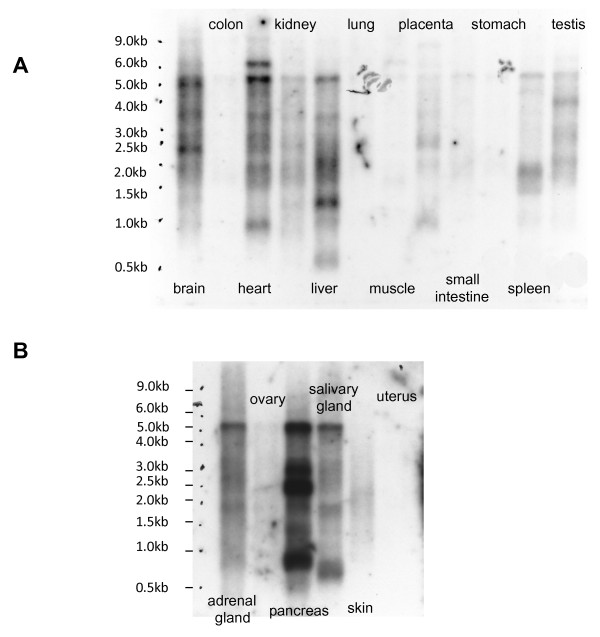
**Detection of HERV-W gag transcripts by Northern blot analysis.** The Northern blot demonstrates that some repetitive elements are highly expressed in brain and other tissues. This is consistent with our whole transcriptome sequencing data where we found a large number of reads mapping to repeat element sequences.

We found a large proportion of mappable reads (an average of 8%) in brain mRNA to originate from repeat sequences. These results are consistent with the findings from a previous report which used the CAGE tagging technology to map repeat elements [23]. We also calculated the number of reads which aligned uniquely to repeat masked intronic and intergenic regions. In our study, the number of reads from intronic and intergenic regions (Additional File 1: Table S1) is much lower than previous estimates for brain expression from these genomic compartments [22]. In addition, the samples sequenced in RUN3 were treated with DNase prior to library construction. Hence, DNA contamination is unlikely to account for our findings. We hypothesized that if repeat elements are present in such abundant amounts in the cortex, then we should be able to detect these elements using Northern blot analysis, a technique which requires high to moderate quantities of the target RNA. We used the human endogenous retrovirus-W (HERV-W) gag sequence as a probe and performed Northern blot analysis on RNA blots containing material from various different human tissues (Figure 1). Several bands are detected in each lane of this blot, consistent with the presence of many HERV-W gag sequences of varying lengths in the genome. These blots confirm that HERV-W gag transcripts are abundantly expressed not only in the brain but also in several other tissue types including pancreas and the heart. Furthermore, the differential banding pattern and intensity of signal between lanes suggests that the expression of these elements is tissue specific.

### Repetitive Element types

**Figure 2:**
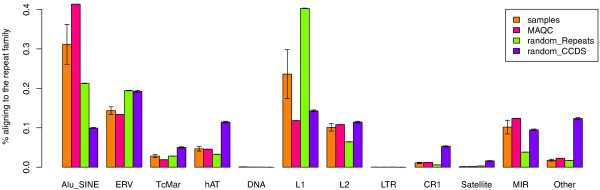
**Distribution of reads by repeat family in the frontal cortex**. Distribution of the reads aligning to the repeats by family observed in the frontal cortex samples and in simulated reads drawn from the database of coding sequences (random CCDS) and from the repetitive sequence (random repeats). Height of the bars corresponds to average frequencies of the reads aligned to a particular family, the error bars indicate plus/minus standard deviation. Averages and standard deviations for samples are derived from pooling the samples, and for simulated data over 100 simulation runs.

To further characterize repetitive element expression, we wanted to determine which types of elements are expressed. Our repeats database contains 141, 145, 063 bp, or about 45% of the genome, as expected. As this database is by definition internally repetitive, we expect that most short reads that align to the database will align to more than one sequence. Instead of attempting to recover all possible alignments for each short read, we chose to keep only one alignment for any read that matched to the database, so that the final counts accumulated along a "consensus" element for each repeat type, though the reads could align to many instances of that repeat in the genome. This strategy prevented us from determining whether any particular genomic regions are more active than others, but enabled us to more confidently determine the expression level of each class of repeats, taken as a group. When we count the reads using this approach, we find a significant amount of Alu, L1/L2, and to a lesser extent, endogenous retrovirus expression in all of the frontal cortex samples (Figure 2). In addition, we downloaded existing RNA sequencing data performed on the human brain from the short reads archive (GSM475204-GSM475209). The distribution of repeat element transcripts in this data, called MAQC, (Additional File 1: Table S1) is very similar to our samples (Figure 2). If repetitive regions were transcribed simply at random, the distribution of short reads mapping to these regions should roughly mirror the percentage of the genome occupied by each repeat class. To determine whether the observed read counts correlated with the number of reads that would be generated at random (and to correct for possible sequence composition-related problems within the data), we created a set of simulated reads from the database of repeat sequences by taking 76 bp subsequences (the size of the original reads) at random from all regions in our repeat database. In this way, we created 100 datasets that contained approximately the same number of repeat-derived reads as what we found from the first two runs of our sequencing data (~400 K short reads). Alignment of each of these 100 sets of simulated short reads to the database of repeats gives the expected outcome if each repeat class in the genome were transcribed at random. These simulations are represented in green bars in Figure 2. According to this analysis, Alu/Sine repeats are expressed at higher levels than expected whereas L1 transcripts are underrepresented. We repeated this analysis for the CCDS database, which contains only expressed sequences, to account for the possibility that the pattern of repetitive expression is driven by repeat elements which are present in coding sequences. For this simulation experiment we sampled 7, 881, 000 reads - an average number of reads produced by the first two sequencing runs for the frontal cortex samples. The result of this analysis is shown in purple bars in Figure 2. The repetitive sequence derived from uniform expression of CCDS annotations can not account for the observed repetitive element expression pattern. However, we know that the sequences in coding regions are not expressed equally and it is quite possible that the high levels of Alu expression may be driven by a single or several Alu sequences which are associated with genes that are expressed in high levels. Since one Alu-derived read can not be mapped specifically to any genomic Alu sequence, this possibility can not be ruled out.

To determine if the differences we found between our simulations, which assumed that repeat element transcription occurred simply at random, and the sequencing data are statistically significant, we used a negative binomial test designed to test for differential expression in the RNA Seq data [25]. Based on this test, the difference between the expected (simulated random expression) and observed transcription pattern for repeat elements is statistically significant (Table 1).

**Table 1:** Significance Tests for Observed Repeat Element Transcription

Repeat Family	Fold Change	p-value	Adjusted for multiple comparisons p-value
Alu_SINE	0.8162648	0.00E+00	0.00E+00

ERV	1.626032	0.00E+00	0.00E+00

TcMar	1.1997436	3.18E-92	4.23E-92

hAT	0.8364789	2.42E-140	3.63E-140

DNA	0.4241692	8.22E-50	9.86E-50

L1	2.1400934	0.00E+00	0.00E+00

L2	0.769354	0.00E+00	0.00E+00

LTR	1.6255619	2.05E-06	2.05E-06

CR1	0.6404078	5.19E-201	1.04E-200

Satellite	2.8788933	2.78E-167	4.77E-167

MIR	0.4463942	0.00E+00	0.00E+00

Other	1.1499157	3.49E-35	3.81E-35

We compared our sequencing data to our simulated reads (simulated reads represent what is expected if repeat element transcription was merely at random) and tested for differential expression. The data was modeled using a negative binomial distribution. The fold changes were derived based on this model. Benjamini-Hochberg method was used to correct for multiple testing. P-values adjusted for multiple testing are shown in the last column.

LINE-1 elements are unique in that when they transpose, the new copy often ends up severely 5' truncated. Only full-length L1 elements are active, so the vast majority of the copies of L1 in the genome are inactive. Upon a careful examination of the 5' end of L1, we observed a clear increase in the number of reads observed from the first few bases of the element, over the simulated data (Additional File 2: figure S1). This indicates that these full-length elements (the only ones capable of retrotransposition) may be expressed at reasonably high levels, though the bulk of the L1 sequences are underexpressed and the 5' end of L1 is the least abundant segment of this element in the genome. Because full length L1 elements carry their own promoter, it is possible that transcripts from these elements are initiated independently from within the L1, not from neighboring genes.

## Discussion

Our results indicate that repetitive elements in the genome are abundantly transcribed in human cortical tissue. A large number of the reads from the sequencing runs aligned to the repeats database we constructed. These results are consistent with the finding of Faulkner et al. [23] but they are much lower than the estimates of Xu et al [22] who report 40% of all transcripts are derived from repetitive sequences. We can not compare our results with Xu et al since they used total RNA as their starting material whereas we used poly-A purified RNA to construct our HTS-libraries. In addition, our study examined cortical tissue which has significantly different gene expression and DNA methylation profiles than the cerebellum [26]. Also, the Northern blot analysis we performed independently validates our findings and assures us that our observations are not due to technical issues related to DNA contamination or peculiarities in the alignment software. Finally, the sequencing data in this paper was generated on two different sequencing platforms, using two separate library preparation techniques by two independent laboratories. The analysis of our sequencing runs along with the MAQC data set yield similar results and demonstrate the robustness of our findings.

The Northern blot analysis we performed suggests that HERV-W gag expression is regulated in a tissue specific manner and these results are consistent with the findings of Nellaker et al [27]. Since the different tissues from the Northern blots did not originate from the same sample, we may be detecting inter individual variation for HERV-W gag expression, which in itself would suggest that this expression is regulated. It is possible, though, that tissue-specific epigenetic differences can drive or repress repeat expression, and this phenomenon can be explored in future studies.

The biological process behind expressing repeat elements at such high levels remains unclear, though random transcription does not explain the expression patterns seen and another mechanism must be involved. The majority of these elements do not have open reading frames (due to mutations) and most likely do not encode proteins, though the full length L1 elements are possibly functional. RNA editing of Alu sequences has been described [28]; the presence of this mechanism suggests that Alu elements are expressed and then suppressed. Other elements have less clear roles; in fact, LINE-1 retrotransposition activity has not been correlated with its expression. Suggesting a possible function for the high repeat expression levels at this point would be purely speculative; here we attempt only to establish a baseline for further analyses.

Despite making up nearly half the human genome, very little is known about the expression patterns of repetitive elements. In this article we report that repetitive elements make up a significant proportion of the cortical transcriptome. In addition to characterizing the tissue-specific expression profile, the inter-individual transcription variation needs to be determined for these elements. Our data indicate inter-individual variation in the expression of repeat element families whereas the total number of repeat elements in the transcriptome of each individual appears to be fairly consistent. Establishing the normal expression state of repeat elements would be particularly useful for determining if alterations to this expression pattern can cause (or indicate) disease. The presence of the RNA editing machinery which appears to target Alu sequences, along with repetitive element expression that is not consistent with a simple "background expression" model suggests that our knowledge of these not-so-silent elements is still incomplete.

## Conclusions

In this article, we report the abundant expression of repeat elements in the human cortex. Remarkably, random background expression can not account for the observed lower than expected LINE-1 and higher than expected Alu element transcription. In addition, our data suggests that full length LINE-1 elements may be expressed at higher levels than truncated copies. Further characterization is required to determine the basis and the consequences of repeat element expression.

## Methods

### Samples

All postmortem brain tissue samples were kindly donated by the Stanley Medical Research Institute. Frontal cortex tissue was dissected from the medial frontal gyrus and total RNA was purified using the RNeasy kit (QIAGEN, CA). The quality of each sample was verified by running an aliquot on a Bioanalyzer (Agilent Technologies, CA).

### Library preparation and sequencing

Three sequencing runs were performed for this paper. RUN1 and RUN2 were performed at Cold Spring Harbor Laboratory and RUN3 was performed at Johns Hopkins University. For the 3 samples in RUN1 and RUN2 10 μg of TRIzol-extracted total RNA was cleaned up using the RNeasy Mini Kit (QIAGEN, CA) prior to cDNA cloning. To isolate the mRNA fraction from each sample, the total RNA was treated to two consecutive rounds of denaturation at 65°C and binding to Dynabeads Oligo(dT)25 (Invitrogen, CA). The purified mRNA samples were then fragmented using Fragmentation Buffer (Ambion, CA) and recovered by ethanol precipitation with glycogen as a carrier. For first strand cDNA synthesis, the mRNA samples were incubated with Random Hexamer Primers (Invitrogen, CA), First Strand Buffer (Invitrogen, CA), RNaseOUT (Invitrogen, CA), DTT, dNTP mix, and SuperScript II (Invitrogen, CA) in a thermal cycler using the following program: 25°C for 10 min, 42°C for 50 min, 70°C for 15 min, and 4°C hold. For second strand cDNA synthesis, the first strand samples were mixed with second strand buffer (50 mM Tris-HCl, pH 7.8; 5 mM MgCl_2_; and 1 mM DTT), dNTP mix, RNase H (Invitrogen, CA), and DNA Polymerase I (Invitrogen, CA), followed with incubation at 16°C for 2.5 h. The cDNA samples were purified using the QIAquick PCR Purification Kit (QIAGEN, CA). Following purification, the samples were converted into cDNA libraries using a series of steps derived from the Paired-End Sample Prep Kit (Illumina, CA). The modifications to the standard library preparation procedure are as follows: (1) after the addition of 3'-A overhangs, the samples were purified using the MinElute PCR Purification Kit (QIAGEN) and the entire eluates were used for the subsequent Illumina adapter ligation step, (2) the selected library fragment size was 200 bp, (3) the gel extraction step was carried out using the MinElute Gel Extraction Kit (QIAGEN, CA) and 20 μL of Buffer EB for elution, and (4) the entire gel extraction eluates were used for PCR enrichment of the libraries using a sixteen-cycle program. The completed cDNA libraries were individually sequenced on an Illumina Genome Analyzer producing 100 base reads from each end. For the 7 samples in RUN3, 5 μg of RNA was treated with Turbo DNase (Ambion, CA) and paired end sequencing libraries were generated using the TruSeq RNA Sample prep kit following the manufacturer's recommended protocol. These libraries were sequenced on a HiSeq 2000 producing 100 base reads from each end.

### Northern blots

Premade blots containing 1 μg of mRNA derived from adult human tissues were purchased from Biochain (Hayward, CA). RNA probes were generated using the Riboprobe kit (Promega, WI) and P-32 labeled cytosines. The hybridization and washes on the blot were carried out using the Northern Max kit (Ambion, CA).

### Reads mapping and simulations

We treated beginnings and ends of the paired-end reads as technical replicates, and aligned them separately. As we accept any alignment of a read to our database of repeats, splitting the paired ends does not compromise our ability to correctly assign reads to repeat families. All reads for the frontal cortex samples were trimmed to 76 base length. We aligned the reads to reference subgenomes (RepBase, intronic, and intergenic regions) using Bowtie software http://bowtie-bio.sourceforge.net/index.shtml[29] using parameters that allow zero mismatches in the first 20 bases of the read, and retain the best alignment for the read. The same parameters were used to align both actual and simulated reads. For alignments to introns and intergenic regions we only accepted reads that aligned exactly once in the entire genome. Genomic coordinates used to build reference subgenomes were determined using UCSC Genome Browser [30] and sequences from hg18 build were fetched using Galaxy (http://g2.bx.psu.edu/)[31].

In simulations we aimed to reproduce the scenario of random (uniform) transcription; while our simulated distributions closely mirror the genomic profiles of the sequences being evaluated, the simulations are critical to establishing statistical significance, as they control for any biases introduced by the alignment process. We extracted genomic sequences for RepBase annotated repeats, generated random start sites for the reads and extracted 76 base pairs for our simulated reads. Sampling from CCDS was done similarly, using consensus coding sequences from NCBI. We generated 100 datasets, each comprising the appropriate number of reads (see Results) and read length.

For statistical testing we pooled the data from the technical replicates of the same biological sample (we considered beginnings and ends of the paired ends to be technical replicates as well). We then compared read counts from these 10 samples mapping to various repeat families with read counts from our simulated data. We employed a statistical test proposed by Anders and Huber [25]. This test for differential expression in RNA-Seq data models the read counts mapping to a given repeat family using a negative binomial distribution with the parameters estimated from the observed data. We used R/Bioconductor package "DESeq" implementation of the test. This R/Bioconductor package performs multiple testing correction using Benjamini-Hochberg method [32].

## Authors' contributions

SS, SW and RY conceived the idea to investigate repeat expression in sequencing data. ST and SW designed the repeats database, performed the alignments and did the statistical analysis. JP constructed the cDNA libraries for RUN1 and RUN2. JP and WM supervised generation of the sequencing data on the Genome Analyzer. MK carried out the initial data analysis. SS constructed the TruSeq cDNA libraries, supervised generation of the HiSeq sequencing data and performed the Northern blot analysis. All authors contributed to the writing of the manuscript. All authors read and approved the final manuscript.

## Supplementary Material

Additional file 1 **Summary of Alignment Results**. Results from aligning each sample to the human genome (build hg18), CCDS database, repeats database, repeat masked intronic and repeat masked intergenic regions. Click here for file

Additional file 2 **The 5 prime end of the L1 element is overrepresented in the sequencing reads (in comparison with the simulated reads)**. Full length L1 elements appear to be expressed at proportions much higher than expected based on the fraction of the genome they compose. For this figure we realigned the reads (both observed and simulated), which mapped to L1s from our repeats database, to the collection of consensus sequences of full length active copies of the L1 elements. We used the LASTZ alignment program [33]http://www.bx.psu.edu/~rsharris/lastz/ instead of Bowtie for this task. Each of the aligning reads usually mapped to several consensus sequences (because they are similar), so we calculated an average base on the L1 where the alignment started. In this plot we show a representative histogram of the distribution of these average starting points for one of the samples and a single draw of simulated reads. Click here for file

## References

[B1] Weber JL May PE Abundant class of human DNA polymorphisms which can be typed using the polymerase chain reaction Am J Hum Genet 1989 44 3 388 388 2916582 PMC1715443

[B2] Richard GF Kerrest A Dujon B Comparative genomics and molecular dynamics of DNA repeats in eukaryotes Microbiol Mol Biol Rev 2008 72 4 686 686 10.1128/MMBR.00011-08 19052325 PMC2593564

[B3] Cordaux R Batzer MA The impact of retrotransposons on human genome evolution Nat Rev Genet 2009 10 10 691 691 10.1038/nrg2640 19763152 PMC2884099

[B4] Armour JA Jeffreys AJ Biology and applications of human minisatellite loci Curr Opin Genet Dev 1992 2 6 850 850 10.1016/S0959-437X(05)80106-6 1477530

[B5] Jern P Coffin JM Effects of retroviruses on host genome function Annu Rev Genet 2008 42 709 732 10.1146/annurev.genet.42.110807.091501 18694346

[B6] Lower R Lower J Kurth R The viruses in all of us: characteristics and biological significance of human endogenous retrovirus sequences Proc Natl Acad Sci USA 1996 93 11 5177 5177 10.1073/pnas.93.11.5177 8643549 PMC39218

[B7] Kazazian HH Goodier JL LINE drive. retrotransposition and genome instability Cell 2002 110 3 277 277 10.1016/S0092-8674(02)00868-1 12176313

[B8] Kazazian HH Wong C Youssoufian H Scott AF Phillips DG Antonarakis SE Haemophilia A resulting from de novo insertion of L1 sequences represents a novel mechanism for mutation in man Nature 1988 332 6160 164 164 10.1038/332164a0 2831458

[B9] Schulz WA Steinhoff C Florl AR Methylation of endogenous human retroelements in health and disease Curr Top Microbiol Immunol 2006 310 211 250 10.1007/3-540-31181-5_11 16909913

[B10] Montoya-Durango DE Liu Y Teneng I Kalbfleisch T Lacy ME Steffen MC Ramos KS Epigenetic control of mammalian LINE-1 retrotransposon by retinoblastoma proteins Mutat Res 2009 665 1-2 20 20 10.1016/j.mrfmmm.2009.02.011 19427507 PMC3418809

[B11] Callinan PA Batzer MA Retrotransposable elements and human disease Genome Dyn 2006 1 104 115 18724056 10.1159/000092503

[B12] Wheelan SJ Aizawa Y Han JS Boeke JD Gene-breaking: a new paradigm for human retrotransposon-mediated gene evolution Genome Res 2005 15 8 1073 1073 10.1101/gr.3688905 16024818 PMC1182219

[B13] Landry JR Mager DL Functional analysis of the endogenous retroviral promoter of the human endothelin B receptor gene J Virol 2003 77 13 7459 7459 10.1128/JVI.77.13.7459-7466.2003 12805445 PMC164795

[B14] Landry JR Medstrand P Mager DL Repetitive elements in the 5' untranslated region of a human zinc-finger gene modulate transcription and translation efficiency Genomics 2001 76 1-3 110 110 10.1006/geno.2001.6604 11549323

[B15] Landry JR Rouhi A Medstrand P Mager DL The Opitz syndrome gene Mid1 is transcribed from a human endogenous retroviral promoter Mol Biol Evol 2002 19 11 1934 1934 12411602 10.1093/oxfordjournals.molbev.a004017

[B16] Balada E Ordi-Ros J Vilardell-Tarres M Molecular mechanisms mediated by human endogenous retroviruses (HERVs) in autoimmunity Rev Med Virol 2009 19 5 273 273 10.1002/rmv.622 19714703

[B17] Conrad B Weissmahr RN Boni J Arcari R Schupbach J Mach B A human endogenous retroviral superantigen as candidate autoimmune gene in type I diabetes Cell 1997 90 2 303 303 10.1016/S0092-8674(00)80338-4 9244304

[B18] Lafon M Jouvin-Marche E Marche PN Perron H Human viral superantigens: to be or not to be transactivated? Trends Immunol 2002 23 5 238 238 10.1016/S1471-4906(02)02207-X 12102742

[B19] Mi S Lee X Li X Veldman GM Finnerty H Racie L LaVallie E Tang XY Edouard P Howes S Syncytin is a captive retroviral envelope protein involved in human placental morphogenesis Nature 2000 403 6771 785 785 10.1038/35001608 10693809

[B20] Weis S Llenos IC Sabunciyan S Dulay JR Isler L Yolken R Perron H Reduced expression of human endogenous retrovirus (HERV)-W GAG protein in the cingulate gyrus and hippocampus in schizophrenia, bipolar disorder, and depression J Neural Transm 2007 114 5 645 645 10.1007/s00702-006-0599-y 17219017

[B21] Frank O Giehl M Zheng C Hehlmann R Leib-Mosch C Seifarth W Human endogenous retrovirus expression profiles in samples from brains of patients with schizophrenia and bipolar disorders J Virol 2005 79 17 10890 10890 10.1128/JVI.79.17.10890-10901.2005 16103141 PMC1193590

[B22] Xu AG He L Li Z Xu Y Li M Fu X Yan Z Yuan Y Menzel C Li N Intergenic and repeat transcription in human, chimpanzee and macaque brains measured by RNA-Seq PLoS Comput Biol 6 e1000843 10.1371/journal.pcbi.1000843 PMC2895644 20617162

[B23] Faulkner GJ Kimura Y Daub CO Wani S Plessy C Irvine KM Schroder K Cloonan N Steptoe AL Lassmann T The regulated retrotransposon transcriptome of mammalian cells Nat Genet 2009 41 5 563 563 10.1038/ng.368 19377475

[B24] Wilhelm BT Marguerat S Watt S Schubert F Wood V Goodhead I Penkett CJ Rogers J Bahler J Dynamic repertoire of a eukaryotic transcriptome surveyed at single-nucleotide resolution Nature 2008 453 7199 1239 1239 10.1038/nature07002 18488015

[B25] Anders S Huber W Differential expression analysis for sequence count data Genome Biol 11 10 R106 10.1186/gb-2010-11-10-r106 PMC3218662 20979621

[B26] Ladd-Acosta C Pevsner J Sabunciyan S Yolken RH Webster MJ Dinkins T Callinan PA Fan JB Potash JB Feinberg AP DNA methylation signatures within the human brain Am J Hum Genet 2007 81 6 1304 1304 10.1086/524110 17999367 PMC2276356

[B27] Nellaker C Li F Uhrzander F Tyrcha J Karlsson H Expression profiling of repetitive elements by melting temperature analysis: variation in HERV-W gag expression across human individuals and tissues BMC Genomics 2009 10 532 10.1186/1471-2164-10-532 19919688 PMC2779825

[B28] Barak M Levanon EY Eisenberg E Paz N Rechavi G Church GM Mehr R Evidence for large diversity in the human transcriptome created by Alu RNA editing Nucleic Acids Res 2009 37 20 6905 6915 10.1093/nar/gkp729 19740767 PMC2777429

[B29] Langmead B Trapnell C Pop M Salzberg SL Ultrafast and memory-efficient alignment of short DNA sequences to the human genome Genome Biol 2009 10 3 R25 10.1186/gb-2009-10-3-r25 19261174 PMC2690996

[B30] Karolchik D Hinrichs AS Furey TS Roskin KM Sugnet CW Haussler D Kent WJ The UCSC Table Browser data retrieval tool Nucleic Acids Res 2004 32 Database D493 D493 14681465 10.1093/nar/gkh103 PMC308837

[B31] Blankenberg D Von Kuster G Coraor N Ananda G Lazarus R Mangan M Nekrutenko A Taylor J Galaxy: a web-based genome analysis tool for experimentalists Curr Protoc Mol Biol Chapter 19 Unit 19 10 11 21 10.1002/0471142727.mb1910s89 PMC4264107 20069535

[B32] Benjamini YHY Controlling the false discovery rate: a practical and powerful approach to multiple testing J Roy Statist Soc Ser B (Methodological) 1995 57 1 289 289

[B33] Harris RS Improved pairwise alignment of genomic DNA 2007 Ph.D. thesis, Pennsylvania State University

