# Supplementary figures and images for: Establishing the baseline level of repetitive element expression in the human cortex
Source: BMC Genomics. 2011 Oct 10;12:495. doi: 10.1186/1471-2164-12-495 (PMC3207997; doi:10.1186/1471-2164-12-495)

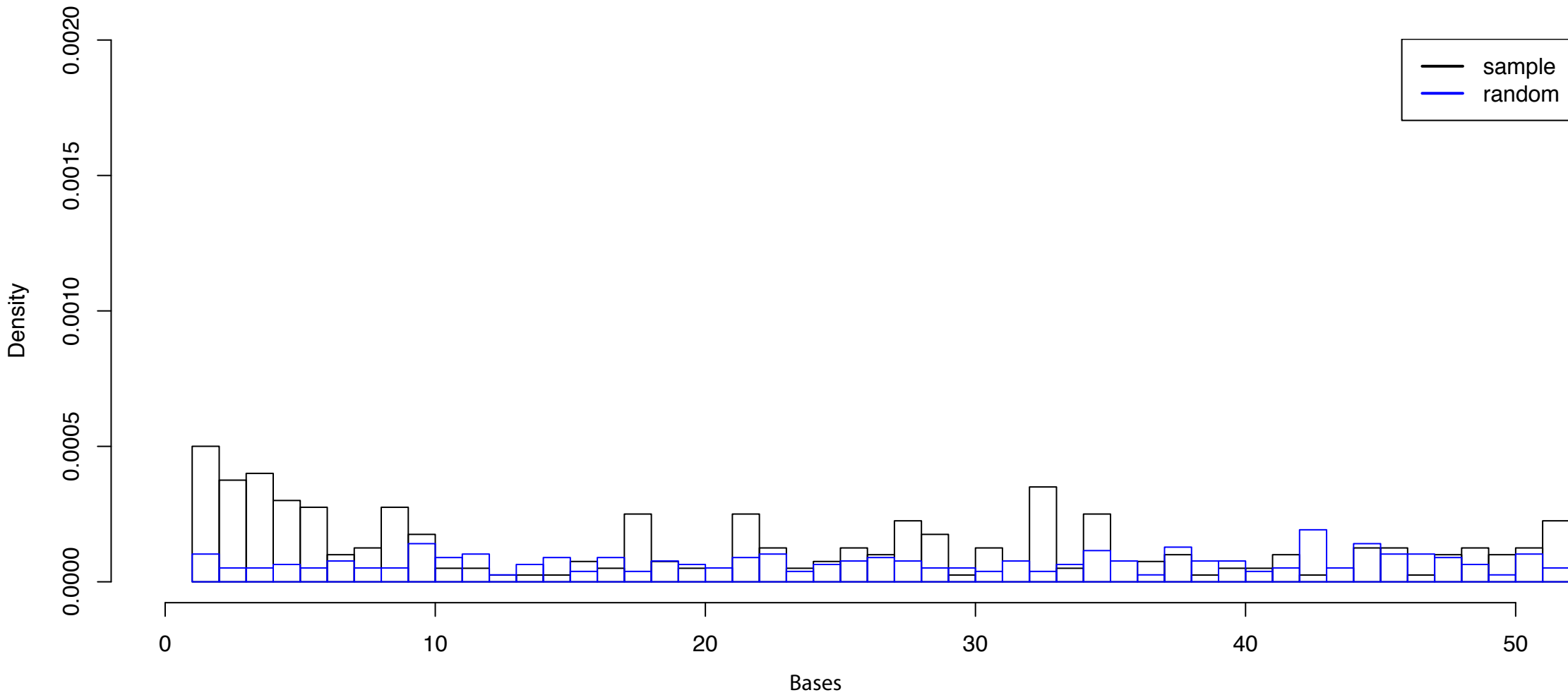

Supplement: Additional file 2 — The 5 prime end of the L1 element is overrepresented in the sequencing reads (in comparison with the simulated reads). Full length L1 elements appear to be expressed at proportions much higher than expected based on the fraction of the genome they compose. For this figure we realigned the reads (both observed and simulated), which mapped to L1s from our repeats database, to the collection of consensus sequences of full length active copies of the L1 elements. We used the LASTZ alignment program [33]http://www.bx.psu.edu/~rsharris/lastz/ instead of Bowtie for this task. Each of the aligning reads usually mapped to several consensus sequences (because they are similar), so we calculated an average base on the L1 where the alignment started. In this plot we show a representative histogram of the distribution of these average starting points for one of the samples and a single draw of simulated reads. [file 1471-2164-12-495-S2.PDF]
